# Meta-analysis of P53 expression and sensitivity to platinum-based chemotherapy in patients with non-small cell lung cancer

**DOI:** 10.1097/MD.0000000000024194

**Published:** 2021-02-05

**Authors:** Sheng Lin, Xiaoqin Li, Ming Lin, WenXiang Yue

**Affiliations:** Department of Respiratory and Critical Care Medicine, Fujian Provincial Hospital,Shengli Clinical Medical College of Fujian Medical University, Dongjie,Road No 134, Fuzhou, Fujian, China.

**Keywords:** chemosensitivity, meta-analysis, non-small cell lung cancer, P53

## Abstract

**Background:**

The relationship between p53 expression and chemosensitivity of non-small cell lung cancer (NSCLC) is unclear. This study aims to explore the correlation between p53 expression and sensitivity to platinum-based chemotherapy in patients with NSCLC.

**Methods:**

Pubmed, Web of Science, EMBASE, CNKI, China Wanfang databases were searched for studies on the relationship between the p53 expression and the chemosensitivity to platinum drugs in patients with NSCLC. The last search time was May 2020. Stata 15.0 software was used for statistical analysis.

**Results:**

A total of 21 studies were included, covering 1387 patients in total. The results showed that the pooled OR = 1.55 (95%CI: 1.05∼2.29, *P* < .05), for Asian population, the pooled OR = 1.67 (95%CI: 0.95∼3.09, *P* > .05), for Caucasian population, the pooled OR = 1.34 (95%CI: 0.74∼2.43), there was no significant difference between Asian and Caucasian. The results of subgroup analysis of publication year showed that, the pooled OR = 2.07 (95%CI: 1.39∼3.07, *P* < .01), the heterogeneity among the studies decreased remarkably after 2005. The subgroup analysis of advanced patients showed that the pooled OR = 1.93 (95%CI: 1.27∼2.93), the difference was statistically significant.

**Conclusion:**

Patients with p53 negative expression is more sensitive to platinum-based chemotherapy than those with p53 positive expression in NSCLC, especially in advanced NSCLC.

## 1 Introduction

Lung cancer, whose cases are increasing rapidly worldwide, is a malignant tumor with the highest mortality rate across the globe.^[1]^ It, due to which the deaths exceed 1 million annually, is also the fastest growing disease in both morbidity and mortality that poses a serious threat to human health.^[1]^ It can be divided into 2 main types based on differences in biological behavior of lung cancer cells: Small Cell Lung Cancer (SCLC) (20%–25%), and Non-small Cell Lung Cancer (NSCLC) (75%–80%). Although the current clinical diagnosis and treatment methods are continuously improving, the 5-year survival rate of NSCLC patients is still less than 15%,^[2]^ and most patients still have recurrence or distant metastasis after radical surgery.^[3]^ Choosing appropriate tumor treatment markers may improve the survival quality and time of patients with NSCLC to some extent. For patients with early-stage NSCLC, surgical treatment is still the optimal choice among various clinical treatments (surgery, chemotherapy and radiotherapy, etc.). However, for patients with advanced NSCLC, surgical eradication is almost impossible, so drug chemotherapy will be the main treatment. For patients with NSCLC, the effect of chemotherapy varies greatly among individuals. There are many reasons for patients with failed chemotherapy, among which drug resistance of tumor cells is the main reason. Most malignant tumors have some similar characteristics when treated with drugs. For instance, patients are relatively sensitive when the drug is used for the first time, but their sensitivity to drug decreases and the amount of drug required will increase during the subsequent use of the drug. This may be due to the drug resistance of tumor cells under various mechanisms during chemotherapy.^[4,5]^

P53 gene is located on chromosome 17pl3.1, containing 11 exons and 10 introns. The length of DNA is 20 kb.^[6]^ Wild-type p53, as a tumor suppressor gene, is a key regulatory protein of cell cycle, which plays a key role in regulating cell proliferation and differentiation. As a “checkpoint” in the G1 phase (pre-synthesis of DNA) in the cell cycle, p53 will check the DNA damage points and monitor the integrity of the genome. If there is a damaged DNA, p53 will stagnate the cell cycle in the G1 phase and determine the “fate” of the cell based on the results of DNA damage and repair.^[7]^ Gene mutations are found in more than 50% of all malignant tumors, and p53 gene mutation or abnormal expression of p53 protein can be detected in 75% of NSCLC.^[8]^ Increased expression of p53 protein can enhance the sensitivity of non-small cell lung cancer to paclitaxel and cisplatin in vitro.^[9]^ P53 mutation can be used as a predictive marker of adjuvant chemotherapy sensitivity in NSCLC.^[10]^

So far, dozens of studies have focused on the relationship between p53 abnormalities and chemosensitivity to platinum drugs in NSCLC, but the conclusions are still not consistent to a great extent. For instance, some studies reported that p53 over-expression increases chemosensitivity to platinum drugs in NSCLC patients,^[11]^ whereas some other studies reported that p53 over expression decreases chemosensitivity to platinum drugs.^[12]^ Meanwhile, several other studies suggested that p53 protein abnormality does not affect chemosensitivity to platinum drugs.^[13]^ Because of differences in experimental methods, sample size, and inconsistency in the research population, it is difficult to generalize the results of a single sample to the entire population. This study conducted a meta-analysis which was designed to reduce the bias and differences among studies, to comprehensively evaluate the results of previous studies to explore the relationship between p53 protein expression and platinum-based chemosensitivity in patients with NSCLC. This study will hopefully provide medical evidence for clinical treatment of lung cancer.

## 2 Materials and methods

### 2.1 Literature retrieval

Pubmed, Web of Science, Excerpta Medica Database (EMBASE), China national knowledge infrastructure (CNKI), China Wanfang databases were searched for studies on the relationship between p53 expression and chemosensitivity of platinum drugs in patients with NSCLC. The retrieval time was from the establishment of databases to May 2020. The retrieval strategy was as follows: (“Non-small cell lung cancer” OR “Lung cancer”) AND (“p53” OR “TP53”) AND (“chemosensitivity” OR “Platinum”). There was no language limitation. Two researchers searched the databases independently, and finally cross-checked the search results. Wherever the 2 researchers encountered disagreements, they should resolve them through discussion.

### 2.2 Inclusion and exclusion criteria

#### 2.2.1 Inclusion criteria

1. Papers should be about the relationship between p53 expression and chemosensitivity to platinum drugs in patients with NSCLC.
2. NSCLC should be the primary lung cancer, diagnosed by operation and pathology, mediastinoscopy, bronchoscopy or puncture biopsy.
3. The criteria for evaluation of the effectiveness of chemotherapy should be provided, with complete remission + partial remission (CR+PR) as the overall response rate.

#### 2.2.2 Exclusion criteria

1. The pathological type of lung cancer was small cell lung cancer.
2. Pathology was diagnosed only by cytology in vitro.
3. P53 protein was detected after chemotherapy or radiotherapy.
4. The study was in vitro cell experiments, animal studies, or only a summary and review.
5. The Newcastle-Ottawa Scale (NOS) score was less than 6.

### 2.3 Data extraction

The 2 researchers extracted data independently and finally cross-checked them. The extracted information was as follows: first author, publication year, country, age, tumor stage, chemotherapy cycle, chemotherapy regimen, median survival time, detection method, cut-off value, the p53 positive and negative cases, and effective cases of chemotherapy of p53 positive or negative expression.

### 2.4 Document quality evaluation

The studies included were evaluated according to NOS.^[14]^ Papers with scores less than 6 stars were of low-quality, and studies with scores equal to or above 6 stars were of high-quality. Only studies with 6 stars and above were selected. The qualities of the studies included were accessed independently by 2 researchers, and finally cross-checked. Wherever the 2 researchers encountered disagreements, they should resolve them through discussion or turn to a third researcher for help.

### 2.5 Statistical methods

The data was analyzed by Stata 15.0 statistical software. OR and its 95%CI were taken as the effect index. Q-test was used to test the heterogeneity among studies. If *I*^2^ ≥ 50%, or *P* ≤ .05, it was considered that there was high heterogeneity, and then a random-effects model (REM) was adopted. If *I*^2^ < 50%, and *P* > .05, it was considered that there was low heterogeneity, and a fixed-effects model (FEM) was used for data merging. Z-test was used to test the significance of the pooled odds ratio (OR) value. Subgroup analyses of ethnicity, tumor stage and publication year were carried out to explore the source of heterogeneity. Publication bias was judged by funnel plot and Egger Test. Finally, sensitivity analysis was conducted to evaluate whether the conclusions were robust or not.

## 3 Results

### 3.1 Results of literature retrieval

**Figure 1:**
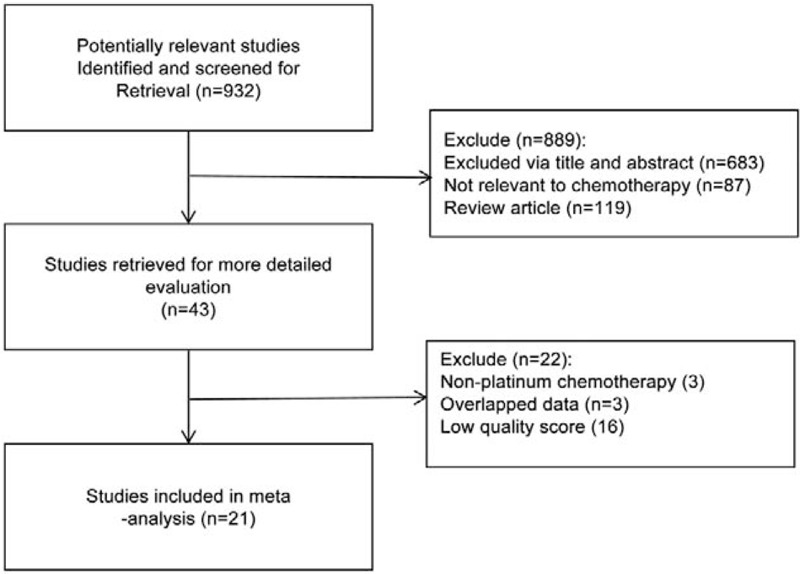
Flow diagram of literature screening.

A total of 21 studies were included in the meta-analysis.^[13–33]^ The specific document screening process was shown in Figure 1. The basic characteristics and quality evaluation of the included studies were displayed in Table 1. There were a total of 1387 patients with NSCLC, including 718 patients with p53 positive expression. The positive expression rate was 51.77%. The NOS scores of the studies included were all equal to or higher than 6.

**Table 1:** Basic characteristics and quality scores of the included studies.

										p53+		p53-		
First author	Year	Country	detection method	Age (years old)	Therapy regimen	Chemotherapy cycle	Median survival (P53+ vs. P53-)(month)	Tumor stage	cut-off value	n	Response rate (%)	n	Response rate (%)	NOS score
Rusch	1995	USA	IHC	NR	Cisplatin-based	2–3	NR	IIIA	5%	28	35.7	24	58.3	8
Nakamishi	1999	Japan	IHC	31–73	cisplatin-based	≥2	NR	III-IV	10%	34	14.7	20	45.0	8
Graziano	2001	USA	IHC	meidan 63	Cisplatin/carboplatin based	2	NR	III	0%	75	26.7	48	33.3	8
Johnson	2002	USA	IHC	NR	Cisplatin-based	1–2	20.9 vs. 20.6	III-IV	5%	25	40.0	24	25.0	8
Gajra	2002	USA	IHC	30–80	Cisplatin/carboplatin based	2	30.7 vs. 40.3	III-IV	1%	50	34.0	39	25.6	8
Cermik	2003	Turkey	IHC	40–79	Cisplatin-based	3	29.7 vs. 46.2	III-IV	5%	7	42.9	8	25.0	8
Harada	2003	Japan	IHC	61.3 ± 10.7	platinum-based	1–6	NR	I-IV	5%	28	42.9	29	17.2	8
Miyatake	2003	Japan	IHC	NR	Cisplatin-based	≥2	4.6 vs. 12.2	III-IV	10%	18	22.2	27	59.3	8
Gregorc	2003	Italy	IHC	38–76	Cisplatin-based	2–3	10.5 vs. 10.5	III-IV	1%	46	26.1	56	57.1	8
Huang PY	2004	China	IHC	18–77	Cisplatin-based	2–4	13.8 vs. 19.9	IIb-IV	25%	17	35.3	28	21.4	7
Liu XG	2005	China	IHC	42–68	Cisplatin-based	3–4	NR	NR	0%	21	76.2	22	40.9	6
Wu HY	2005	China	IHC	38–72	Cisplatin-based	2–3	15 vs. 18	III-IV	10%	20	40.0	18	55.6	7
Zhang R	2005	China	IHC	NR	Cisplatin-based	≥2	NR	III-IV	0%	72	25.0	69	56.5	6
Fijolek	2006	Poland	IHC	41–73	Cisplatin-based	2	NR	II-III	5%	16	31.3	51	43.1	8
Hao P	2009	China	IHC	30–73	Cisplatin-based	2–4	11.4 vs. 14.5	II-IV	0%	41	39.0	33	63.6	7
Kaira	2011	Japan	IHC	45–77	platinum-based	NR	19.1 vs. 34.8	IA-IIIB	10%	28	28.6	28	35.7	8
Ju F	2012	China	IHC	35–72	Cisplatin-based	≥2	NR	IV	10%	25	64.0	23	52.2	7
Wang Y	2015	China	IHC	63.2∼68.2	Cisplatin/carboplatin based	3–4	14 vs. 18	III-IV	50%	34	23.5	39	53.8	7
Rashed	2017	Egypt	IHC	25–77	carboplatin based	NR	7 vs. 12.5	III-IV	50%	25	44.0	25	56.0	8
Zheng RF	2017	China	IHC	NR	platinum-based	NR	NR	III-IV	10%	47	29.8	39	53.8	7
Zhang XS	2018	China	IHC	≥65	Cisplatin-based	NR	NR	NR	50%	61	52.5	19	78.9	8

### 3.2 Relationship between P53 expression and chemosensitivity of platinum drugs in NSCLC

**Figure 2:**
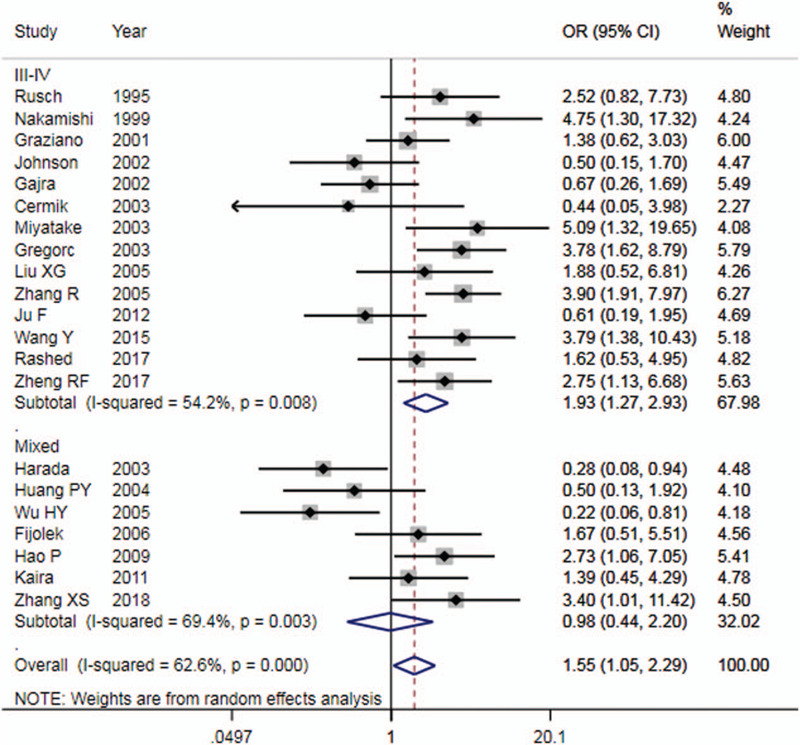
Forest plot of p53 expression and platinum chemosensitivity in non-small cell lung cancer. The upper part of the figure showed the data of studies including only late-stage patients; the lower part of the figure showed the data of studies including all-stage patients.

The main results of this meta-analysis were shown in Table 2. There was statistical heterogeneity (*I*^2^ = 62.6%) among studies, so a random-effects model was used for analysis. Forest plot was shown in Figure 2. The results showed that compared with patients with p53 positive expression, patients with p53 negative expression were more sensitive to platinum-based chemotherapy, the pooled OR = 1.55 (95%CI: 1.05∼2.29, *P* < .05).

**Table 2:** Main results of Meta-analysis of p53 and chemosensitivity in patients with NSCLC.

chemosensitivity	Subgroup	n	OR	95%CI	*P*	*I*^2^	*P* for heterogeneity	Model	*P* (Egger)
Overall		21	1.55	1.05∼2.29	.028	62.6	.000	REM	.165
Ethnicity	Caucasian	7	1.34	0.74∼2.43	.329	52.6	.049	REM	.486
	Asian	13	1.67	0.95∼2.91	.073	69.7	.000	REM	.178
Published year	Before 2005	13	1.28	0.71∼2.32	.410	72.8	.000	REM	.322
	After 2005	8	2.07	1.39∼3.07	.000	10.1	.352	FEM	.259
Tumor stage	III-IV	14	1.93	1.27∼2.93	.002	54.2	.008	REM	.536

The results of ethnicity subgroup analysis showed that, for the Asian population, there was no significant difference, the pooled OR = 1.67 (95%CI: 0.95∼2.91, *P* > .05); for the Caucasian population, there was no significant difference, the pooled OR = 1.34 (95%CI: 0.74∼2.43). Nevertheless, there was no significant decrease in heterogeneity either in Asians or Caucasians.

The results of subgroup analysis of publication year showed that, the heterogeneity among studies published after 2005 decreased significantly (I^2^ = 10.1%, *P* > .05), OR = 2.07 (95%CI: 1.39∼3.07, *P* < .05). There were significant statistical differences.

The subgroup analysis of patients with advanced NSCLC (Tumor stage III-IV) showed that, NSCLC patients with p53 negative expression were more sensitive to platinum chemotherapy than those with p53 positive expression, the pooled OR = 1.93 (95%CI: 1.27∼2.93, *P* < .01). Moreover, it was also observed that the heterogeneity among studies in advanced NSCLC was low. This suggested that there was a correlation between p53 protein expression and chemosensitivity of platinum drugs in patients with advanced NSCLC, and the chemosensitivity of patients with negative expression of p53 protein was higher than that of patients with positive expression of p53 protein, especially in advanced NSCLC.

### 3.3 Publication bias analysis

**Figure 3:**
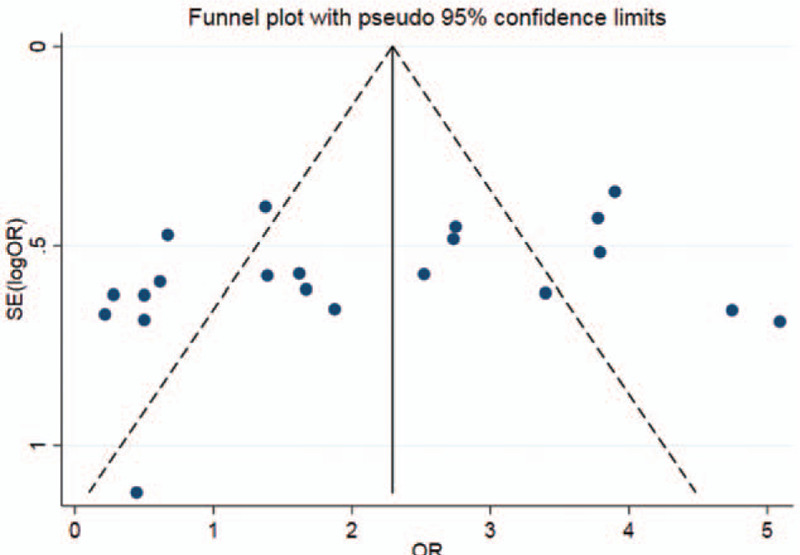
Funnel plot of p53 expression and platinum chemosensitivity in non-small cell lung cancer.

Publication bias was detected by funnel plot and Egger Test. The funnel plot (Fig. 3) was basically symmetrical, and Egger Test (Table 2) showed that there was no publication bias.

### 3.4 Sensitivity analysis

**Figure 4:**
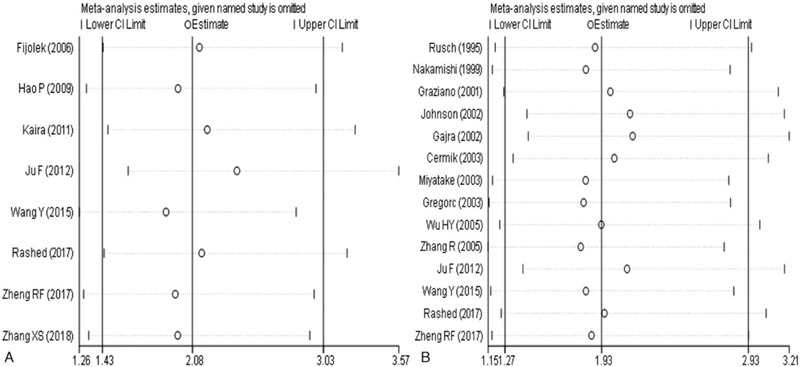
Sensitivity analysis of p53 expression and platinum chemosensitivity in non-small cell lung cancer (A: sensitivity analysis of the overall study; B: Sensitivity Analysis of the studies after 2005).

The results of sensitivity analysis were displayed in Figure 4. Each study was excluded one by one, and then a meta-analysis was conducted again to access the impact of each study to the overall result. The sensitivity analysis of the research in advanced NSCLC showed that there were no statistically significant changes in the combined effects after eliminating any single study (Fig. 4B). The sensitivity analysis of the research after 2005 showed that there was no statistically significant change in the results after the exclusion of any single study (Fig. 4A). This proofed the conclusions we reached were robust.

## 4 Discussion

There are controversies among studies upon the relationship between p53 protein expression and chemotherapy sensitivity to platinum drugs in NSCLC. Therefore, we conducted this meta-analysis. The results showed that there was a strong correlation between p53 protein expression and chemosensitivity to platinum drugs in NSCLC. P53 expression played an essential role in the occurrence and development of lung cancer. Zhao et al^[34]^ reported that microRNA-374b is able to mediate the occurrence of NSCLC by regulating expression of ITGB1 and p53. Hsa-miR-217 is also able to inhibit the proliferation, migration and invasion of NSCLC cells by targeting p53/KAI1 signal.^[35]^ Park et al^[36]^ suggested that PBK reduces paclitaxel-induced autophagy cell death by inhibiting p53 in NSCLC cells. In vitro experiments, sensitivity of lung cancer cells to platinum-based chemotherapy is closely related to expression of P53. Feldmann et al^[37]^ proved that nanoparticles-carrying siRNA enhances sensitivity of platinum in the model of p53 wild-type NSCLC. P53 would also make chemically resistant non-small cell lung cancer sensitive by increasing the reactive oxygen species and inhibiting EGFR/PI3K/AKT signal.^[38]^ Moreover, in advanced NSCLC, p53 mutations are strongly associated with immune checkpoint inhibitor response and longer survival.^[39]^

When all studies were enrolled in the meta-analysis of p53 protein expression and platinum-based chemosensitivity in patients with NSCLC, it was found that, OR = 1.55, the difference was statistically significant. Nonetheless, the heterogeneity among studies was high. Hence, we conducted subgroup analyses respectively to explore the source of heterogeneity. According to the subgroup analysis of ethnicity, it was found that there was no significant decrease in heterogeneity between the 2 groups. According to the subgroup analysis of publication year, it was found that the heterogeneity among studies increased before 2005, whereas decreased significantly after 2005. The results showed that, OR = 2.07, the difference was statistically significant. Subgroup analysis of patients with advanced NSCLC showed that the difference was statistically significant. OR = 1.93, the heterogeneity decreased significantly. Sensitivity analysis confirmed that this conclusion was robust. Therefore, the conclusions drawn from the subgroup analysis after 2005 and in advanced NSCLC were convincing. A meta-analysis by Wang et al^[40]^ in 2008 showed that there is no correlation between p53 protein expression and platinum-based chemosensitivity in patients with advanced NSCLC. But different conclusions were reached in our study, which may be due to our inclusion of more high-quality studies.

Certainly, this study had some limitations. Firstly, when all the studies were included in the analysis, there was a large heterogeneity. After carrying out subgroup analyses of ethnicity and publication year, it was found that the publication year and tumor stage were important sources of heterogeneity which was yet little impacted by ethnicity. However, other sources of heterogeneity such as age stratification and cut-off value were not further presented in our study. Secondly, although the conclusion of p53 expression and chemosensitivity to platinum drugs in advanced NSCLC was confirmed in our study, relatively few studies were included and the sample size was small, which may lead to bias to some extent. Thirdly, immunohistochemical method was mainly applied in this study. However, different antibody manufacturers, item numbers and batches had a great impact on the experimental results. Besides, different studies would set different cut-off values, which may also bring some bias to the results.

In conclusion, there is a certain correlation between p53 protein expression and chemosensitivity of platinum drugs in patients with NSCLC, especially in patients with advanced NSCLC. The platinum-based chemosensitivity of patients with p53 negative expression is higher than that of patients with p53 positive expression. It is the first time that the correlation between p53 expression and sensitivity of platinum-based chemotherapy has been confirmed by a meta-analysis in patients with NSCLC. Nonetheless, in view of the limitations of this study, it is necessary to explore the relationship between the 2 to a deeper extent in the future.

## Author contributions

Sheng Lin, WenXiang Yue: Critical revision of the manuscript; Sheng Lin, Xiaoqin Li and WenXiang Yue: Substantial contribution to the conception and design of the work, manuscript drafting; Xiaoqin Li, Ming Lin: Acquisition, analysis, and interpretation of the data; Sheng Lin, WenXiang Yue: Revising the manuscript critically, final approval of the version to be published. All authors have read and approved the final manuscript.

**Conceptualization:** Sheng Lin, Xiaoqin Li, WenXiang Yue.

**Data curation:** Sheng Lin, Xiaoqin Li, Ming Lin.

**Formal analysis:** Sheng Lin, Xiaoqin Li, Ming Lin.

**Funding acquisition:** Sheng Lin.

**Investigation:** Sheng Lin, Xiaoqin Li, Ming Lin.

**Methodology:** Sheng Lin, Ming Lin, WenXiang Yue.

**Project administration:** Sheng Lin, WenXiang Yue.

**Resources:** Sheng Lin, Xiaoqin Li.

**Software:** Sheng Lin, Xiaoqin Li, Ming Lin.

**Supervision:** Sheng Lin, Ming Lin, WenXiang Yue.

**Validation:** Sheng Lin, Xiaoqin Li, Ming Lin.

**Visualization:** Sheng Lin, Xiaoqin Li.

**Writing – original draft:** Sheng Lin, Xiaoqin Li, Ming Lin.

**Writing – review & editing:** Sheng Lin, Xiaoqin Li, Ming Lin, WenXiang Yue.
